# A Large-Scale Initiative Inviting Patients to Share Personal Fitness Tracker Data with Their Providers: Initial Results

**DOI:** 10.1371/journal.pone.0165908

**Published:** 2016-11-15

**Authors:** Joshua M. Pevnick, Garth Fuller, Ray Duncan, Brennan M. R. Spiegel

**Affiliations:** 1 Division of General Internal Medicine, Department of Medicine, Cedars-Sinai Medical Center, 8700 Beverly Blvd, Los Angeles, California, United States of America; 2 Division of Informatics, Department of Biomedical Sciences, 8700 Beverly Blvd, Los Angeles, California, United States of America; 3 Center for Outcomes Research and Education, Cedars-Sinai Medical Center, 8700 Beverly Blvd, Los Angeles, California, United States of America; Utrecht University, NETHERLANDS

## Abstract

**Background:**

Personal fitness trackers (PFT) have substantial potential to improve healthcare.

**Objective:**

To quantify and characterize early adopters who shared their PFT data with providers.

**Methods:**

We used bivariate statistics and logistic regression to compare patients who shared any PFT data vs. patients who did not.

**Results:**

A patient portal was used to invite 79,953 registered portal users to share their data. Of 66,105 users included in our analysis, 499 (0.8%) uploaded data during an initial 37-day study period. Bivariate and regression analysis showed that early adopters were more likely than non-adopters to be younger, male, white, health system employees, and to have higher BMIs. Neither comorbidities nor utilization predicted adoption.

**Conclusion:**

Our results demonstrate that patients had little intrinsic desire to share PFT data with their providers, and suggest that patients most at risk for poor health outcomes are least likely to share PFT data. Marketing, incentives, and/or cultural change may be needed to induce such data-sharing.

## Background and Significance

In late 2014, 64% of US adults owned smartphones.[1] Most new smartphones can be used with or without complementary PFTs to help automate acquisition of biometric data, including step and stair counts, body weight, heart rate, and blood pressure. Furthermore, standalone PFTs are increasingly used by the general public to track biometric data. Such data has incredible potential for characterizing, tracking, and ultimately improving patients’ health.[2] However, one small study suggested that people with chronic illnesses were much less likely to use such devices, as compared to healthy people.[3] We sought to evaluate the willingness of large populations to share PFT data with healthcare providers via their electronic health records (EHR), which might allow providers to leverage this data to provide better healthcare. After inviting 79,953 patients to sync their PFTs to an EHR for continuous uploading of PFT data, we compared patients who synced their PFTs with those who did not. To develop *a priori* hypotheses and guide our comparison of demographic, health, and socioeconomic characteristics of both groups of patients, we consulted the Extended Unified Theory of Acceptance and Use of Technology [4] and existing literature on health information technology and patient portal adoption.

## Methods

This study utilized the patient portal of CS-Link, Cedars-Sinai Health System’s (CSHS) branding of the EpicCare enterprise EHR product (Epic Systems Corporation, Verona, WI). CS-Link is used at a large, non-profit hospital and at many local associated provider organizations both within and outside CSHS. These organizations provide multidisciplinary care across the care continuum to a socioeconomically diverse population.

To move towards an organizational goal of driving patient engagement and use of an existing patient portal, CSHS began inviting patients to sync PFTs on April 25, 2015. All registered patient portal users received a message entitled “Syncing your Wearable Devices With My CS-Link” stating “You can now sync data from your own personal health devices with your Cedars-Sinai medical record using My CS-Link^TM^. Click HERE to learn more.” Users also received an email indicating a new portal message. All applications and devices compatible with the Apple HealthKit, Fitbit, and Withings software frameworks were supported.

We began our analysis by reviewing the literature to generate *a priori* hypotheses regarding which patient characteristics would predict device syncing.[4–11] Because we found few studies of PFT adoption, we also extrapolated from study of health information technology in general, and of patient portals specifically. Our review suggested that compared to non-adopters, patients choosing to share PFT data would be more likely to be younger, white, non-Hispanic, English-speaking, insured, higher income, employed, sicker, and higher utilizers of healthcare.

Next, we determined which patients had uploaded PFT data via synced devices by June 1, 2015. We queried the EHR and other electronic sources of administrative data to obtain the necessary data elements. We excluded patients without any portal logins during January 1, 2015–April 24, 2015, patients not 18–89 years old, and patients with at least one missing or clearly erroneous data element. We used bivariate statistics to compare characteristics of patients who uploaded any data vs. patients who did not. We then constructed a logistic regression model using complete case analysis (Stata, College Station, TX). The analysis received no explicit external funding. The CSHS Institutional Review Board approved this analysis with a waiver of informed consent based on its use of de-identified data.

## Results

Of 79,953 registered portal users invited to sync devices over the 37 days of our study period, we excluded: 8019 users without any portal logins during January 1, 2015–April 24, 2015; 513 users over 89 years old; 2809 users under 18 years old; and 2507 users with missing or clearly erroneous data. Of 66,105 remaining users, 499 (0.8%) uploaded data during the first 37 days of this initiative. Bivariate analysis showed that these early adopters were more likely than non-adopters to be younger, male, and white, lower income, CSHS employees, and to have higher body mass indices (BMI). Each aforementioned association except income was robust to multivariable regression, which also showed early adopters were more likely to be insured and non-Hispanic (**Table 1**). Neither Charlson comorbidity index nor CSHS utilization (as measured by cost, hospitalizations, or outpatient encounters) predicted adoption.

**Table 1:** Demographic, Health, and Socioeconomic Characteristics of 66,105 Patients Invited to Upload Personal Fitness Tracker Data

Characteristic*	Early Adopters (n = 499)			Non-Adopters (n = 65,606)		*P* Value for Difference	Odds Ratio from Multivariable Model	*P* Value
**Demographic**								
Mean age (SD), *y*	44.4		(12.2)	48.9	(15.8)	<0.001^†^	0.98 (0.97–0.98)	<0.001
Female sex, *n (%)*	252		(51)	38,373	(58)	<0.001^‡^	0.67 (0.25–0.80)	<0.001
Hispanic, *n (%)*	47		(9)	6,450	(10)	0.10	0.61 (0.44–0.83)	0.003
Race, *n (%)*						0.69		
White	351		(70)	44,514	(68)		1.37^§^ (1.13–1.70)	0.002
Black	46		(9)	6,613	(10)			
Asian	46		(9)	6,697	(10)			
Other	56		(11)	7,782	(12)			
English as 1^st^ language, *n (%)*	482		(97)	62,120	(95)	0.06	1.46 (0.89–2.40)	0.24
Health system employee, *n (%)*	85		(17)	5,123	(8)	<0.001	2.50 (1.95–3.21)	<0.001
**Health**								
Mean Body Mass Index (SD)	27.9		(5.7)	26.3	(5.3)	<0.001	1.06 (1.04–1.07)	<0.001
Mean Charlson comorbidity score (SD)	0.14		(0.62)	0.22	(0.92)	0.07	0.94 (0.82–1.07)	0.30
**Socioeconomic**								
Health insurance, *n (%)*		476	(95)	61,319	(93)	0.08	1.82 (1.20–2.79)	0.006
Mean annual income (SD), US dollars**		69,283	(26,709)	71,991	(29,304)	0.04	0.99 (0.99–1.00)	0.46

* We measured and tested utilization in several ways, but found it not to be associated with adoption.

† Continuous variables subjected to two-tailed T-test.

‡ Categorical variables subjected to Chi-square test.

^§^ Odds ratio for adopters being white vs non-white.

** Estimated from median annual incomes in zip code of residence, using 2010 US Census data.

## Discussion

In this large-scale initiative inviting patient portal users to sync PFTs with an EHR, only 0.8% of users uploaded data. This low uptake was likely related to the use of only one invitation via patient portal. Indeed, greater uploading among organizational employees suggests that greater awareness of the initiative would have induced more adoption. To be sure, this might also reflect a better understanding of the potential utility of such data or greater confidence in the ability of the institution to hold such data private. Nonetheless, the low adoption rate demonstrates that there was not substantial intrinsic desire to share PFT data, such that marketing, incentives, and/or cultural change may be needed to increase syncing. Although population-level data suggests that the majority of the patient portal user population likely had access to a compatible device, [1] addressing users’ willingness and ability to sync devices would likely increase data sharing.

Regarding health status, early adopters were younger and without greater comorbidity or resource utilization, but had higher BMIs. If these higher BMIs were due to adipose tissue stores rather than muscle mass, these higher BMIs may reflect greater potential for health benefit among early adopters. However, our other results, except for the higher adoption rate among males, suggest that those most at risk for poor health outcomes were least likely to share PFT data with providers. A systematic review of patient portals also found decreased use among racial and ethnic minorities.[9] However, it found that higher portal use was associated with worse health status, which contrasts with our finding of less PFT data sharing among those with worse health status.

The Extended Unified Theory of Acceptance and Use of Technology could help to explain such differences, in that hedonic motivation may be responsible for early adoption of PFT data sharing among younger, healthier men.[4] In contrast, effort expectancy, facilitating conditions, social influence, and/or performance expectancy may drive longer term use of patient portals among sicker patients. If this is the case, we should expect to see increased sharing of PFT data among sicker patients as this technology matures. Future research should test this hypothesis by studying data sharing over a longer time period. Other improvements might involve more active recruitment strategies, which could involve multiple communications and specific information regarding the value of sharing PFT data with providers. Researchers should also seek to understand patients’ reluctance to share PFT data, to increase such sharing, and to measure its benefits, especially among those at risk for poor health outcomes.
